# Hedgehog Signaling in Prostate Cancer and Its Therapeutic Implication

**DOI:** 10.3390/ijms140713979

**Published:** 2013-07-04

**Authors:** Annelies Gonnissen, Sofie Isebaert, Karin Haustermans

**Affiliations:** Laboratory of Experimental Radiotherapy, Department of Oncology, KU Leuven, & Radiation Oncology, University Hospitals Leuven, Herestraat 49, 3000 Leuven, Belgium; E-Mails: annelies.gonnissen@med.kuleuven.be (A.G.); sofie.isebaert@med.kuleuven.be (S.I.)

**Keywords:** hedgehog pathway, prostate cancer, combination treatment, radiotherapy, chemotherapy, molecular targeted agents

## Abstract

Activation of Hedgehog (Hh) signaling is implicated in the development and progression of several tumor types, including prostate cancer, which is still the most common non-skin malignancy and the third leading cause of cancer-related mortality in men in industrialized countries worldwide. Several studies have indicated that the Hh pathway plays a crucial role in the development as well as in the progression of this disease to more aggressive and even therapy-resistant disease states. Moreover, preclinical data have shown that inhibition of Hh signaling has the potential to reduce prostate cancer invasiveness and metastatic potential. Clinical trials investigating the benefit of Hh inhibitors in patients with prostate cancer have recently been initiated. However, acquired drug resistance has already been observed in other tumor types after long-term Hh inhibition. Therefore, combining Hh inhibitors with ionizing radiation, chemotherapy or other molecular targeted agents could represent an alternative therapeutic strategy. In this review, we will highlight the role of Hh signaling in the development and progression of prostate cancer and summarize the different therapeutic applications of Hedgehog inhibition.

## 1. Introduction

The Hedgehog (Hh) signaling pathway is essential for numerous processes during embryonic development including cell growth, cell differentiation, patterning and organogenesis. In normal adult tissues, this pathway is involved in stem cell population maintenance, tissue repair and regeneration [1–4]. In various types of cancer on the other hand, uncontrolled activation of the Hh signaling pathway has been observed [2,5–7]. As for prostate cancer (PCa), there is emerging evidence that Hh signaling plays a crucial role in the development as well as in the progression of this disease to more aggressive and even therapy-resistant disease states [1].

In this review, we will highlight the role of Hh signaling in the development and progression of PCa and summarize the different therapeutic applications of Hh inhibition.

## 2. Hedgehog Signaling

### 2.1. Hedgehog Signaling Pathway

The Hh pathway consists of a very complex signaling network that is still being unraveled [2,5,6]. According to the most recent model, canonical pathway activation is initiated by peptide ligands, called hedgehogs. In humans, three homologous Hh ligands exist: Sonic hedgehog (Shh), Indian hedgehog (Ihh) and Desert Hedgehog (Dhh), of which Shh is the best studied. These ligands are synthesized as precursor proteins that subsequently undergo several posttranslational modifications, including autocatalytic cleavage and lipid modifications, which are necessary for the proper secretion and reception of the ligands. Secretion of the mature ligand from the producing cell is further mediated by the membrane protein Dispatched. On the cell surface of the receiving cell, both negative (e.g., Hhip) as well as positive (e.g., Cdo) regulators are expressed, which respectively compete with [8] or enhance the binding of the Hh ligand to its receptor [9,10], the twelve-span transmembrane receptor Patched (Ptch). Humans encode two homologs of this receptor protein, Ptch1 and Ptch2, with similar affinity for Hh ligands and differential expression in various tissues [11].

Ptch is a rather unusual receptor because it functions as a pathway inhibitor, blocking pathway activation in the absence of Hh ligand by inhibiting the seven-span transmembrane protein Smoothened (Smo) [12]. Smo exists in an inactive and an active state that appears to be defined, besides other modifications, by its location within the cell, *i.e.*, inside or outside the primary cilia [13]. Primary cilia are cell surface protrusions found on most, if not all, vertebrate cells that function as sensory “antennae” for signal transduction [14]. These organelles seem to be of crucial importance for Hh signaling since all the major pathway components localize herein [13,15].

In the absence of Hh, Ptch localizes to the base of the primary cilium and prevents the movement of Smo from the plasma membrane and endoplasmic vesicles into the primary cilium. This restricted access of Smo to the primary cilium is released upon Hh ligand binding to Ptch, resulting in accumulation of Smo in the cilium [16,17]. Complete activation of Smo however requires a secondary, currently incompletely understood, activation step that is probably also regulated by Ptch [18]. The presence of active Smo in the tip of the cilium induces a functional change in the organelle that fundamentally alters the manner in which the members of the Gli family of transcription factors (Gli1, Gli2, Gli3) are post-translationally processed, involving protein phosphorylation, proteasome-mediated proteolysis and cytoplasmic-nuclear shuttling. Gli1 only occurs as a full-length transcriptional activator (Gli-A), while Gli2 and mainly Gli3 can be processed into truncated repressor forms (Gli-R) [19,20]. The presence of activated Smo within the primary cilium suppresses the generation of Gli-R forms [21].

A major negative regulator of Gli activity is the Suppressor of Fused (Sufu). Sufu binds to all three Glis and controls the processing and/or degradation of Gli and thereby the Gli-A:Gli-R ratio [2]. Sufu may also act more downstream in controlling the cytoplasmic-nuclear shuttling of Gli [22]. The means by which ligand binding counteracts Sufu’s repression of Gli activity is still unclear, but could involve ubiquitin/proteasome-mediated degradation of Sufu [23].

The best documented target genes of active Gli transcription include *GLI1* and *PTCH1*, of which the corresponding proteins are important positive and negative regulators of the pathway itself. One function of the transcriptional output is thus to establish feedback loops to control Hh pathway activity. Other verified target genes include cell cycle regulators (e.g., *CYCLIN D1/2*, *N-MYC*), anti-apoptotic molecules (e.g., *BCL2*), angiogenic molecules (e.g., *VEGF*, *ANG1-2*), epithelial-mesenchymal transition (EMT) regulators (e.g., *SNAIL*, *MMP9*), molecules implicated in self-renewal and cell fate determination (e.g., *NANOG*, *OCT4*, *SOX2*) as well as effectors of other developmental signaling pathways (e.g., Wnt) [24].

Besides the classical Hh signal transduction, non-canonical Smo-independent pathway activation may also occur. There seems to be significant crosstalk of Hh signaling with important oncogenic pathways such as the MAPK, PI3K, NFκB and TGF-β pathways as well as with the key tumor suppressor molecules p53 and PTEN [24–26].

### 2.2. Hedgehog Signaling in Cancer

In recent years, it has become increasingly clear that aberrant Hh signaling plays a major role in cancer initiation and progression to more advanced stages. This was discovered for the first time in 1960 in patients with Gorlin syndrome, a rare hereditary condition whereby patients develop several basal cell carcinomas (BCC) and medulloblastomas (MB) during their lifetime. This disease is mainly caused by mutations in the *PTCH1* gene, but also mutations in *SMO* and *SUFU* have been described [27,28]. Meanwhile, activated Hh signaling has been demonstrated in more than 30% of human cancers, including basal cell carcinoma, medulloblastoma, lymphoma, leukemia, ovarian, breast, pancreatic, lung, liver, gastrointestinal, prostate and bladder cancer [29].

Hh pathway activation in cancer can be categorized in four principle models: (**a**) ligand-independent signaling; (**b**) ligand-dependent autocrine signaling; (**c**) ligand-dependent paracrine signaling and (**d**) ligand-dependent reverse paracrine signaling. However, these signaling types are not mutually exclusive but can also co-exist [2,30,31]. Ligand-independent Hh signaling has mainly been described in BCC and MB and can either be due to loss-of-function mutations (*PTCH1*, *SUFU*) or otherwise caused by gain-of-function mutations (*SHH*, *SMO* or *GLI1/2*). Aberrant Hh signaling in the other tumor types is generally caused by ligand-dependent Hh activation, but still a lot of controversy exists about whether this is due to autocrine or paracrine signaling or due to a combination of both. Initially, it was thought that ligand-dependent Hh signaling occurred in an autocrine manner, whereby the tumor cell produces the Hh ligands and causes cell-autonomous Hh pathway activation. This was based on the fact that both Hh ligands as well as downstream Hh signaling components were expressed in the tumor cells and that the growth of these cells could be inhibited *in vitro* with cyclopamine in the absence of a stromal compartment [31]. However, in several tumor types (e.g., pancreas, prostate, ovarian and colorectal), Hh pathway activation was rather present in the adjacent stroma than in the tumor itself suggesting the presence of paracrine signaling [32–34]. As for the latter, tumor cells produce Hh ligands and signal to the surrounding stroma, which in turn causes the production of factors that indirectly stimulate tumor progression. Furthermore, Scales *et al.* described a variant of paracrine signaling whereby signaling occurs in the opposite direction, so-called reverse paracrine signaling [31]. This model was based on observations in B-cell malignancies in which Hh ligands were secreted from the bone marrow stroma leading to stimulation of tumor survival and growth [35,36].

Another important aspect of Hh signaling is the role of the primary cilia, which can act as both positive and negative regulators of the Hh pathway. On the one hand, primary cilia are crucial for the activation of Hh signaling, since the translocation of Smo to the primary cilium is essential to activate the Gli transcription factors. On the other hand, primary cilia are also critical for the proteolytic processing of Gli3 to its repressor form (Gli-R), which occurs in the absence of Hh ligand [37]. Therefore, depending on where exactly in the Hh signaling pathway the brake has been removed, primary cilia may be necessary for pathway activation or not [38–40]. Disruption of the primary cilia in Smo-activated tumors inhibits tumor growth, whereas tumor growth is accelerated in Gli2-dependent tumors [38,39]. Furthermore, when taking into account the above mentioned models for Hh pathway activation, it seems that ligand-dependent signaling pathway activation can only be cilia-dependent, whereas ligand-independent (mutation-driven) pathway activation can be either cilia-dependent or cilia-independent [40].

### 2.3. Hedgehog Signaling in Prostate Cancer

Hh signaling plays an essential role in the embryonic development of the prostate. Hh signaling is actively present in the epithelium of the urogenital sinus from where the prostate derives [41]. During prostate development, Hh signaling mainly functions in the ductal budding and ductal extension, but is also important for tissue polarity [42–44]. In the adult prostate, Hh signaling is relatively low but still present and important for regeneration of prostate epithelium [43].

**Figure 1:**
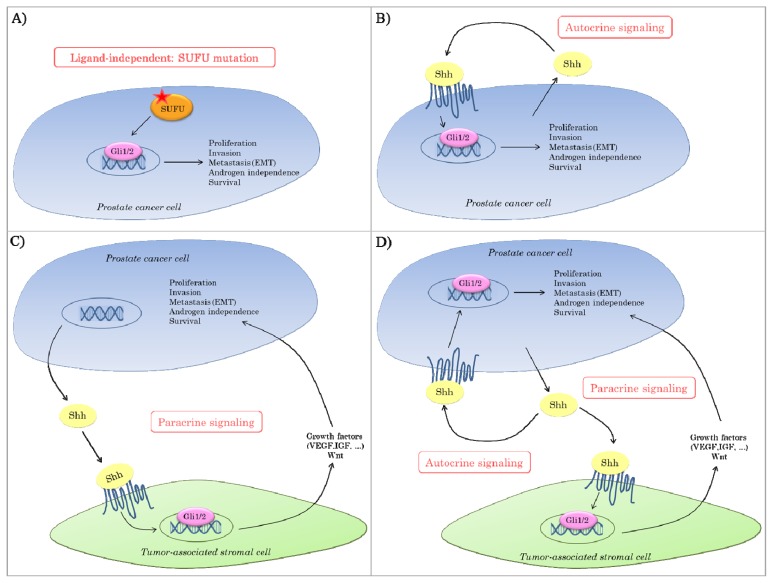
Modes of aberrant Hedgehog (Hh) signaling in prostate cancer. Hh signaling can be activated by different mechanisms. (**A**) Ligand-independent Hh signaling caused by a mutation in *SUFU*; (**B**) ligand-dependent autocrine signaling; (**C**) ligand-dependent paracrine signaling or (**D**) combined ligand-dependent autocrine and paracrine signaling.

Increasing evidence suggests an active role for Hh signaling in the development and progression of PCa. However, there is still a lot of controversy about the exact mode of aberrant Hh signaling in PCa (Figure 1). Multiple components of the Hh pathway are present within chromosomal regions associated with susceptibility to human PCa. Nevertheless, loss-of function mutations in *SUFU* are the only known mutations in the Hh pathway in prostatic tumor tissues thus far (Figure 1A) [45,46]. In general, however, aberrant Hh signaling in prostate tumors is believed to be ligand-dependent. As described above, it remains controversial whether this is mediated in a paracrine and/or autocrine manner. On the one hand, it has been reported that Hh ligands produced by tumor cells signal to the tumor-surrounding stroma, thereby inducing the production of growth factors that support tumor growth and/or survival (Figure 1C) [34]. On the other hand, there are data suggesting that the tumor switches to an autocrine requirement for Hh signaling in which the tumor cells both produce and respond to the ligand (Figure 1B) [1,45,47]. It could also be that in some cases paracrine and autocrine mechanisms co-exist, so that Hh overexpression by the tumor cells orchestrates effective tumor growth by direct stimulation of tumor cell proliferation in an environment rich in supporting survival and angiogenic factors (Figure 1D). If this is the case, Hh antagonists might be particularly effective since these could suppress Hh signaling both in the prostate tumor as well as in its microenvironment.

Notwithstanding the above mentioned discussion, several studies on prostatic tissue from patients with PCa have demonstrated Hh signaling activity (Table 1), suggesting an active role for this pathway in PCa [1,34,45,47–50]. Tzelepi and colleagues have evaluated the protein expression of different Hh components (Shh, Ptch, Smo and Gli1) in tissue microarrays constructed with 141 prostatic tumor tissue samples, 119 adjacent non-neoplastic peripheral zone (PZ) tissue samples and 53 bone marrow PCa metastases tissue samples. Tumor epithelial expression of Shh, Smo and Ptch was up-regulated compared to the non-neoplastic epithelium, whereas stromal Ptch, Smo and Gli1 were down-regulated in the tumor tissue [48]. Sanchez *et al.* examined the protein expression of Shh on tissue microarrays representing 239 prostate carcinomas, 15 precancerous high-grade prostatic intraepithelial neoplasias and 135 benign prostate tissues. They found higher Shh expression to be more often present in tumors (33%) compared to normal adjacent tissue (<1%), with higher Shh levels correlating with increased proliferation (Ki67) [47]. In a study by Fan *et al.*, gene expression of *SHH* and *GLI1* was compared between 6 prostate tumor tissue samples, 6 benign prostatic hyperplasia (BPH) samples and 7 benign prostatic tissue samples. In contrast to the previous studies, statistical analysis revealed no significant differences in expression between the different types of prostatic tissue [34]. This could be explained by the bulk extraction from tumors that was used in this study for quantitative RT-PCR, resulting in comixtures of tumor with benign stromal cells. Therefore, immunohistochemical analysis could represent a more suitable approach for these kinds of analyses [51].

Furthermore, Hh pathway activation seems to be more pronounced in advanced PCa. Sheng *et al.* reported that high levels of Ptch1 and Hedgehog-interacting protein (Hhip) were more frequently detected in PCa with high Gleason score and metastatic PCa specimens [45]. Moreover, Tzepeli *et al.* demonstrated that expression of Ptch in the tumor tissue correlated with tumor grade and stage. Epithelial Ptch expression was also found to be higher in metastatic tissue compared to primary PCa tissue. Moreover, Hh signaling also correlated with Ki67 and vascular epithelial growth factor (VEGF), but not with CD31 [48]. The group of Azoulay *et al.* [49] evaluated Hh ligand expression in 231 hormone-naïve (HNPC), 20 hormone-treated (HTPC) and 24 hormone-refractory (HRPC) prostate tumor samples. In HNPC, a significant correlation was found between Shh expression and Gleason score on the one hand and metastasis in the lymph nodes on the other hand. Likewise, epithelial Dhh expression was significantly associated with Gleason score, tumor stage and seminal vesicle invasion. Multivariate analysis also presented the concomitant absence of Shh and Dhh in stromal cells as an independent prognostic parameter for biological recurrence in PCa [49]. A study by Kim *et al.* [50] showed that Hh signaling is associated with poor prognosis. In this retrospective study of 155 PCa samples, the protein expression of different Hh signaling components (Shh, Ptch, Smo, Gli and Sufu) was examined and correlated with clinicopathological parameters, including tumor size, Gleason score, pretreatment PSA and PSA recurrence. All the investigated Hh components except Sufu were significantly correlated with Gleason score. Furthermore, Shh expression was found to be a significant independent prognostic factor for PSA recurrence in multivariate analysis [50]. Karhadkar *et al.* compared the gene expression of Hh ligands and Hh transcriptional targets *PTCH1* and *GLI1* between localized and metastatic prostate tissue. *SHH* and *IHH* were present in all samples, being either benign prostate tissue, localized or metastatic prostate tumor tissues. However, while *PTCH1* and *GLI1* were expressed in all metastatic tumor samples, only 3 out of 12 localized tumor samples and none of the benign tissue samples expressed these genes. Moreover, *PTCH1* mRNA levels were more than tenfold higher in metastatic tissue compared to localized PCa samples. Furthermore, the authors showed that transfection of a poorly metastatic cell line (AT2.1) with Gli1 increased the metastatic potential of this cell line remarkably, illustrating the role of Hh signaling in promoting metastasis [1]. In order to investigate the progression of PCa and the development of metastasis, proper representative animal models of PCa are necessary. For PCa, two different mouse models have been described; the LADY PCa mouse model and the TRAMP PCa model. An *in vivo* study in the LADY PCa mouse model by Gipp and colleagues has shown that the expression of Hh signaling components, Shh, Ptch1 and Gli1 are not increased during PCa development [52], whereas another study by Bragina *et al.* using a TRAMP PCa mouse model did show an age-dependent increase in Hh activity associated with tumor development [53]. These contradictory results could be linked to the differences between the two tumor models. The LADY mice develop rather low-grade prostatic intraepithelial neoplasia and invasive carcinoma, which generally fail to metastasize, whereas TRAMP mice are more advanced and able to metastasize primarily to lungs and lymph nodes [53].

In addition, a potential relationship between Hh signaling and androgen-independent PCa has been described by multiple independent groups. Long-term androgen deprivation has been shown to induce an up-regulation of Hh signaling both in human specimens [49,54,55] and in PCa cell lines [49,54–57], suggesting an active role for Hh signaling in the progression to androgen-independent PCa. For instance, Efstathiou *et al.* reported that different Hh signaling components (Shh, Smo, Gli1 and Gli2) were increased after androgen deprivation therapy (ADT) compared to untreated control samples, both in human as in mouse xenograft samples. Moreover, combination of ADT and chemotherapy also resulted in an increased epithelial Bcl2 and nuclear pAKT expression, emphasizing the role of Hh signaling activation in tumor progression [54]. A recent study by Ibuki and colleagues demonstrated that the Hh inhibitor, TAK-441 was able to delay the progression to castration-resistant PCa (CRPCa) in a PCa xenograft mouse model. However, in this study, TAK-441 had no effect on cell viability of LNCaP cells *in vitro* after androgen withdrawal, indicating that the effect of Hh inhibition on tumor progression is probably due to paracrine Hh signaling in the surrounding tumor stroma [55]. This is in contrast with a study by Chen *et al.*, which showed that inhibition of Hh signaling in the absence of androgens resulted in a decrease of LNCaP cell growth *in vitro* and this effect was rescued by the addition of androgens to the medium. In addition, inhibition of Hh signaling led to down-regulation of androgen receptor (AR) signaling activity, at least partly due to direct binding of Gli2 and/or Gli1 to the AR [58,59]. Shaw *et al.* investigated the effect of concomitant inhibition of Hh signaling and ErbB signaling in CRPCa cells *in vitro.* The Hh and ErbB pathways seemed to have synergistic effects on the proliferation of CRPCa cells, resulting in a more pronounced inhibition of CRPCa cell growth [60]. Nevertheless, more investigation is needed to gain more insight into the exact mechanisms behind the involvement of Hh signaling in the progression to CRPCa.

The above mentioned data suggest an active role for Hh signaling in the initiation and/or progression of PCa, however, the exact mechanisms how Hh signaling regulates these processes are not completely understood. Thiyagarajan *et al.* reported that Gli2 is actively involved in the malignant transformation of PCa. Knockout of GLI2 in PCa cells suppressed tumor growth both *in vitro* and *in vivo*. The mechanism behind this was ascribed to the effect of Gli2 on the cell cycle. Ectopic expression of GLI2 led to an accelerated cell cycle progression, especially through the G2-M phase, consequently resulting in an increased cell growth [61]. Another study by Chung *et al.* indicated that Hh signaling could function through stathmin1. Stathmin1 is a microtubule-regulating protein that is important in the assembly and disassembly of the mitotic spindle. Inhibition of Hh signaling reduced the expression of stathmin1 in PCa cells, whereas recombinant Shh increased stathmin1 expression. Inhibition of Hh signaling and stathmin1 both decreased PCa cell proliferation, but no additive effect was observed, indicating that Hh signaling presumably functions through regulation of stathmin1. The effect of Hh signaling on cell cycle progression could hence be through modulation of stathmin1 and thus of the assembly of the mitotic spindle [62]. Moreover, the role of Hh signaling in the initiation and progression of PCa has, at least partly, also been ascribed to its anti-apoptotic properties and its effects on invasiveness and metastasis [63]. Several studies have demonstrated that inhibition of Hh signaling induces apoptosis of PCa cells both *in vitro* and *in vivo* [64,65]. Nanta *et al.* indicated that Hh inhibition resulted in suppression of EMT, as illustrated by decreased cell motility, invasion and migration of PCa cells after treatment with the Smo inhibitor NVP-LDE-225 [65]. The Hh pathway itself is also regulated by several mechanisms. For instance, a study by McKee *et al.* demonstrated that Hh signaling is regulated by protease nexin 1 (PN1), a serine protease inhibitor present in the extracellular matrix (ECM), which is normally expressed in the prostate. PN1 suppresses Hh signaling activity in the prostate by decreasing the level of Shh ligand. The level of PN1 itself is negatively regulated by matrix metalloproteinase 9 (MMP9), which is frequently up-regulated in human malignancies and associated with tumor progression, invasion and metastasis. Thus, increased levels of MMP9 inhibit PN1 function in PCa, thereby leading to an elevated Hh signaling activity and hence PCa progression [66].

In summary, Hh signaling seems to be involved in the development of PCa as well as in the progression to more aggressive and even therapy-resistant disease states. Hence, targeting Hh signaling pathway could represent a valuable treatment option for PCa, which is still the most common non-skin malignancy and the third leading cause of cancer-related mortality in men in Europe [67] and in developed countries worldwide [68]. Especially for high-risk PCa patients, *i.e.*, those who are at high risk for PCa recurrence and dying from their disease, new treatment strategies are warranted. Since 20%–35% of all newly-diagnosed PCa patients are classified as high-risk, successful inhibition of Hh signaling in PCa could potentially have a major impact on the management of this disease [69,70].

## 3. Therapeutic Application of Hedgehog Inhibition

### 3.1. Hh Inhibitors

The discovery and development of agents that are able to regulate the activity of the Hh pathway is a very rapidly expanding field. A detailed description of all Hh inhibitors is beyond the scope of this review, and we kindly refer the reader to some excellent reviews on this topic [71,72]. In short, both natural compounds as well as synthetic molecules are available that either target upstream pathway components (e.g., Shh, Smo) or block the last steps of the pathway, *i.e.*, the Gli transcription factors [71,72]. The most extensively studied compound is cyclopamine, a naturally occurring molecule derived from the plant Veratrum californicum. Cyclopamine inhibits Hh signaling by acting on Smo with an EC50 of approximately 300 nM [65,66]. Preclinical studies in MB and BCC with cyclopamine were very promising, but this compound failed further development in clinical trials due to its poor pharmacokinetic characteristics (highly insoluble in water, poor chemical stability in acidic conditions), low potency and associated toxicity [67,68]. This prompted the development of small-molecule Hh pathway modulators with improved potency and druggability. Nowadays, numerous Hh pathway inhibitors have already been developed, most of them targeting Smo, but also small molecules against Gli1/2 (GANT58, GANT61) and Shh (Robotnikinin) are currently under preclinical development in various tumor types, including PCa.

As stated above, the presence or absence of primary cilia is an important aspect of Hh signaling activity, depending on nature of the initiating oncogenic event being either up- or downstream of the cilia [38,40]. This implies that the effectiveness of targeting different steps of the Hh pathway could be influenced by the expression level of these cilia. For example, treating patients who have a Hh ligand-driven cancer would be predicted to be effective only if the tumor cells are ciliated. Emerging data, however, suggest that cilia dysfunction is a common event in cancer. When the cilia are lost, high Hh ligand levels are no longer relevant and a secondary mutation downstream of the cilia (e.g., inactivating mutation of SUFU) would be required to sustain pathway activation. In this case, it will be necessary to target inhibition of the Hh pathway downstream of the cilia. A combination of Hh-targeted drugs that are both cilia-dependent as well as cilia-independent could potentially overcome the resistance due to tumor heterogeneity in terms of cilia frequency. Much research is still needed to determine whether the predicted relationships between the presence of cilia and responsiveness to specific Hh pathway inhibitors are clinically relevant [40].

### 3.2. Hh Signaling Inhibition as Monotherapy

Hh inhibitors seem to be highly efficient in ligand-independent Hh activated tumors, *i.e.*, BCC and MB. At present, (clinical) investigations are ongoing to evaluate their potential efficacy in a variety of ligand-dependent cancer types (e.g., prostate, pancreas, ovarian cancer) [30]. As for the treatment of PCa, several preclinical studies have shown that inhibition of Hh signaling reduces tumor growth as well as PCa invasiveness and metastatic potential [1,47]. Karhadkar *et al.* have shown that cyclopamine inhibits tumor growth of PCa cell lines both *in vitro* and *in vivo* [1]. Datta *et al.* even reported a complete prostate tumor regression that remained in remission for 70–148 days post-treatment with this drug [46]. Furthermore, a recent study by Karlou *et al.* indicated that GDC-0449 also inhibits tumor proliferation of PCa xenograft mice. GDC-0449 inhibited gene expression of *PTCH1* and *GLI1.* Moreover, a reduction in proliferation was seen after GDC-0449 treatment by means of decreased Ki67 expression level; however, no change in tumor volume was observed [73].

Despite the promising preclinical results of Hh inhibition as monotherapy in PCa, this has not been translated into the clinic. A phase I clinical trial testing the use of the GDC-0449 as a single agent in patients with BCC, MB and other advanced solid tumor types, including PCa, reported a complete or partial tumor response in patients with BCC (19/33) and MB (1/1). Unfortunately, no response was observed in patients with other tumor types, such as PCa [74]. Since this study only included two PCa patients, phase II clinical trials, testing the use of Hh inhibitors as monotherapy are warranted to demonstrate its efficacy in PCa patients. Meanwhile, the efficacy of Hh inhibition in patients with advanced BCC or MB has been demonstrated in two phase II clinical trials [75,76]. This led to the approval of vismodegib, also known as GDC-0449, by Food and Drug Administration (FDA) for treatment of locally advanced and metastatic BCCs. Other Smo inhibitors (LDE-225, TAK-441, PF-04449913, IPI-926, BMS-833923, LY2940680, LEQ506 itraconazole and vitamin D3) are currently being evaluated in clinical trials for the treatment of MB, BCC and other advanced tumor types, including PCa [77]. Hh inhibitors targeting other components of the Hh pathway are still under preclinical investigation.

Hedgehog pathway inhibitors seem to have an acceptable toxicity profile. The main side effects of Smo inhibitors consistently seen in clinical trials are muscle spasms, dysgeusia, fatigue, alopecia and nausea [78]. Although these toxicities are generally characterized as mild, their chronic and persistent nature led to the discontinuation of 30%–54% of patients in two phase II clinical trials testing GDC-0449 treatment in patients with MB and BCC [75,76]. Due to its essential role during embryonic development, Hh inhibitors are contraindicated during pregnancy as these are potentially teratogenic, embryotoxic and fetotoxic [74]. Additionally, the use of Hh inhibitors in young children is not recommended because of skeletal growth complications, including effects on both cartilage and bone formation [79].

Unfortunately, although vismodegib and other Smo inhibitors initially appeared effective, resistance to Smo inhibition has already been identified in patients with MB and BCC during treatment with vismodegib [80,81]. At the moment, it cannot be distinguished if this resistance is due to drug-mediated selection of pre-existing resistant subpopulations or if resistance is acquired due to drug-induced changes that render the cells resistant [82]. Acquired resistance to Smo inhibition has been linked to distinct mechanisms, such as mutations in *SMO* (e.g., D473H) [83], amplifications of downstream target genes (e.g., *GLI1/2*) or up-regulation of synergistic signals such as PI3K signaling [82,84]. Treating these patients with second-generation antagonists such as Smo antagonists that are still effective in vismodegib-resistant patients (e.g., HhAntag), Smo antagonists with a different mechanism of action (e.g., itraconazole) [85] or Hh pathway antagonists more downstream of Smo (e.g., GANT61) could be a potential solution. Another way to overcome this resistance could be by means of combined therapy with ionizing radiation, chemotherapy or with other molecular targeted therapies.

### 3.3. Hedgehog Inhibitors in Combination with Radiotherapy

**Figure 2:**
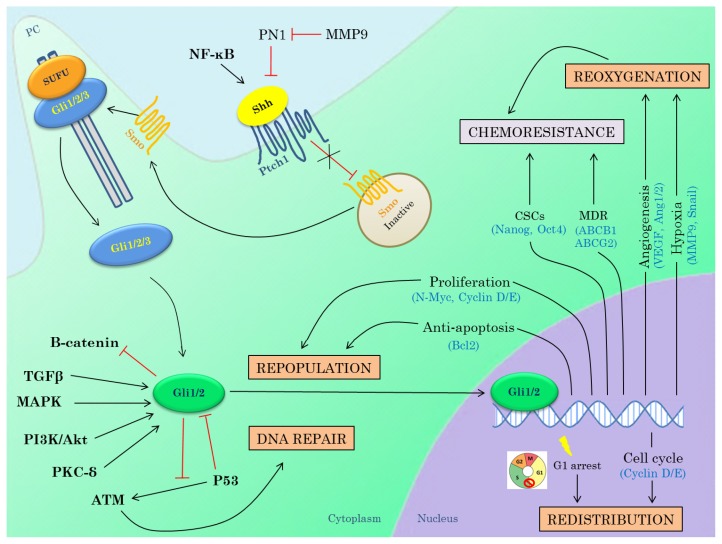
Schematic overview of Hedgehog signaling and rationale for combination therapy with (chemo)radiotherapy. Upon Sonic Hedgehog (Shh) ligand binding to its receptor Patched (Ptch1) 1, the repression of Smoothened (Smo) is relieved, resulting in the movement of Smo from the intracellular vesicles to the primary cilium. Smo becomes activated and promotes the activation of the Gli proteins (Gli1/2) that enter the nucleus and promote transcription of the target genes (canonical pathway activation). The Gli transcription factors can also become activated by means of non-canonical pathway activation due to significant crosstalk with other important pathways such as the PI3K-Akt, KRAS, PKC-δ and TGFβ pathways. The Hh signaling also has important interactions with Wnt pathway and P53. The response to radiation therapy is determined by the four R’s of radiobiology: repopulation, repair of sublethal DNA damage, redistribution and reoxygenation. Hh signaling can potentially interfere with all these processes and targeting Hh signaling could therefore increase radiosensitivity of tumor cells. Moreover, inhibition of Hh signaling could also improve the response to chemotherapy by targeting multidrug resistance and cancer stems cells in addition to its effects on tumor vasculature. Abbreviations: PC, primary cilia; MDR, multidrug resistance; CSCs, cancer stem cells.

The response to radiation therapy is generally determined by the four R’s of radiobiology: repopulation, repair of sublethal DNA damage, redistribution in the cell cycle and reoxygenation of hypoxic regions. Targeting the Hh pathway could potentially affect all of these mechanisms, since Hh signaling regulates the transcription of many genes involved in these processes, Hh pathway activation could result in radiation resistance and therefore this could increase radiosensitivity of tumor cells (Figure 2).

There are several indications that the Hh pathway itself could be a potential target for radiosensitization. First, multiple preclinical studies have demonstrated that Hh signaling is involved in radiation resistance. Chen *et al.* have shown that in hepatocellular carcinomas, soluble factors such as Shh are secreted in the medium in response to ionizing radiation (IR), which resulted in radioprotection. Moreover, mRNA expression of Hh pathway target gene *PTCH1* was increased by IR, Shh ligand stimulation and their combination, indicating that Hh signaling was activated. Antibody neutralization of Shh ligand or knockdown of Gli1 blocked the radioprotective effect [86]. Inhibition of Hh signaling in esophageal, pancreatic and non-small cell lung cancer cell lines, either chemically or through siRNA-mediated silencing also resulted in radiosensitization [87–89].

Second, several clinical studies have indicated that Hh signaling activation after chemoradiotherapy is associated with poor outcome. In a study by Sims-Mourtada *et al.*, esophageal cancer specimens were obtained from 43 patients with esophageal cancer who received neoadjuvant chemoradiotherapy (CRT) prior to surgery. Immunohistochemical analysis of Shh and nuclear Gli1 expression in residual tumors revealed that the Hh pathway was extensively activated in the majority (36/43) of these chemoradiotherapy-resistant tumors [87]. Yoshikawa *et al.* correlated Gli1 expression with clinicopathological parameters (lymph node and distant metastasis, disease-free survival (DFS) and overall survival (OS)) in esophageal cancer specimens after CRT. All patients with nuclear Gli1 expression had lymph node and/or distant metastasis. Moreover, Kaplan–Meier analysis indicated that nuclear Gli1 expression was associated with a significantly decreased DFS and OS [90]. In a similar study by Zhu *et al.*, the expression of Gli1 and Ptch1 was determined in esophageal cancer patients treated with preoperative CRT and correlated with different clinicopathological parameters. Besides Gli1, Ptch1 was also indicated to be an independent prognostic factor for locoregional progression-free survival (PFS), distant PFS and OS [91]. Furthermore, Chaudary and colleagues correlated the gene expression of different Hh components (*SHH*, *IHH*, *PTCH1*, *PTCH2*, *GLI1*) with clinicopathological data (tumor hypoxia, local recurrence and DFS) from cervical adenocarcinoma samples after CRT. The expression of Hh genes was very high in cervical cancer after CRT, whereas up-regulation of *SMO* was associated with local recurrence [92]. Despite the fact that all these data demonstrate a clear link between Hh signaling and radioresistance, the combination of Hh inhibitors with radiotherapy has not been investigated in clinical studies so far.

#### 3.3.1. DNA Repair

Hh signaling components (e.g., Ptch1, Gli1/2) have been linked to genomic instability, inactivation of homologous recombination (HR), non-homologous end joining (NHEJ) and defects in checkpoint activation [93,94]. A study by Mazumdar *et al.* demonstrated that both pharmacological (GANT61) and genetic (Gli3-R) inhibition of Hh signaling in colon carcinoma cells induced DNA damage and cell death. More specifically, the results indicated that an ATM-Chk2-dependent DNA damage response was induced within 24 h upon treatment. Moreover, these effects were more pronounced when targeting the Hh pathway downstream of Smo, since cyclopamine showed little effects on DNA damage, indicating that this is probably due to non-canonical Hh pathway activation [95]. Additionally, the association between Hh signaling and potentially lethal damage repair (PLDR) has been established by Shafaee *et al.* in pancreatic cancer cells [88]. Furthermore, activation of Shh may impair the early DNA damage repair and thereby protect human cancer cells against IR in an autocrine manner [86].

#### 3.3.2. Repopulation

Hh signaling has been suggested to promote tumor repopulation after chemoradiotherapy and to contribute to chemoradiation resistance. Sims-Mourtada and colleagues have examined the relationship between Hh signaling and proliferation after chemoradiotherapy in esophageal adenocarcinoma xenografts. Here, it was shown that 6 to 8 days after chemoradiotherapy, an increase in tumor proliferation was preceded by an increased Hh signaling activity, suggesting an active role of Hh signaling in repopulation [87]. Hh signaling could also promote repopulation after IR by its effects on the transcription of mitogenic and anti-apoptotic genes (e.g., *CYCLIN D1/2*, *N-MYC*, *BCL2*).

#### 3.3.3. Redistribution

Another determinant of the cell’s sensitivity to radiotherapy is the cell cycle phase, with cells being most radiosensitive in the G2M phase, less sensitive in the G1 phase, and least sensitive during the late S phase [96]. Gli1 regulates the transcription of multiple genes that control the cell cycle distribution (e.g., *CYCLIN D1/2*, *N-MYC*). In addition, it was shown by Sims-Mourtada *et al.* that inhibition of Hh signaling in combination with IR results in G1 arrest, thereby decreasing the number of cells in the radioresistant S phase of the cell cycle [87].

#### 3.3.4. Reoxygenation

A link has been described between Hh signaling and hypoxia, of which the latter is known to contribute to tumor metastasis and (radiation) therapy resistance [97]. Preclinical data in cancer cell lines and animal models have shown that hypoxia is able to activate the Hh signaling pathway both in a ligand-dependent [98,99] as well as in a ligand-independent manner by upregulation of Smo transcription [97]. To our knowledge, this link has not been investigated in human (PCa) samples. In a study from Onishi *et al.*, it was demonstrated that hypoxia activates the Hh signaling pathway in pancreatic cancer cells in a ligand-independent manner by up-regulation of Smo transcription. Up-regulation of Smo increased transcription of Gli1 and MMP9 which led to increased tumor cell invasiveness. Moreover, immunohistochemical stainings in human pancreatic tumor samples revealed a significant correlation between Smo, Gli1 and MMP9 expression and the hypoxia marker CA9. These data indicated that the Hh pathway could be a valuable target to counteract the hypoxia-induced invasiveness [97].

Additionally, Hh signaling seems to be associated with the induction of neo-angiogenesis to sustain tumor growth and metastasis. Hh signaling regulates the transcription of pro-angiogenic (e.g., *ANG1/2*, *VEGF*) and EMT regulating genes (e.g., *MMP9*, *SNAIL*) [100,101]. Moreover, Hh inhibition with cyclopamine or IPI-926) increased tumor perfusion through depletion of the tumor-associated stromal tissue, which resulted in more effective delivery of chemotherapeutic agents [100,102,103]. Very recent work from McKee *et al.* indicates a new link between Hh signaling and angiogenesis in PCa. Protease nexin 1 (PN1), a serine protease inhibitor present in the ECM, which is normally expressed in the prostate, seems to regulate proliferation, angiogenesis and invasion of PCa cells through inhibition of Hh signaling [66,104]. PN1 inhibits Hh signaling by reducing the level of the ligand Shh and is able to decrease proliferation of PCa cell lines, both *in vitro* and *in vivo*. PN1 expression in under control of MMP9 and thereby indirectly regulates Hh signaling. In a PCa xenograft mouse model, they have shown that combined treatment of Hh inhibitor GDC-0449 and recombinant PN1 protein altered tumor vasculature, *i.e.*, fewer vessels and larger overall diameter and ultimately even resulted in tumor regression [66,104].

#### 3.3.5. Interactions between Hh Pathway and Genes Known to Induce Radioresistance

In addition to the potential radiosensitizing mechanisms described above, Hh signaling also interacts with other important oncogenic pathways, known to be implicated in radioresistance. The RAS/MEK/ERK and PI3K/Akt pathways are well known to be involved in mechanisms of radioresistance [105,106]. Both RAS/MEK/ERK and PI3K/Akt pathways affect DNA repair after IR, respectively by regulating the transcription of DNA repair genes (e.g., *XRCC1*, *RAD51*, *ATM*) and controlling the activity of kinase activity repair genes (DNA-PK) [107]. Moreover, both pathways contribute to repopulation during radiation treatment due to their pro-proliferative (RAS/MEK/ERK) and anti-apoptotic (PI3K/Akt) effects [108].

Multiple lines of evidence support the interaction between Hh signaling and RAS/MEK/ERK and PI3K/Akt pathways. Stecca *et al.* have demonstrated that Gli1 function is enhanced by RAS/MEK/ERK and PI3K/Akt pathways by enhancing the nuclear localization and transcriptional activity in melanomas [109]. This is in line with research of Riobo and colleagues who have shown that PI3K/Akt and MEK1 stimulate Gli1 activity in NIH 3T3 cells [110,111]. Additionally, Ji *et al.* indicated that KRAS suppresses Gli1 degradation in pancreatic cancer cells [112].

Moreover, crosstalk between Hh signaling and the tumor suppressor gene *P53* is described. Alterations in *P53* are detected in more than 50% of human cancers [113], including PCa [114,115]. The role of p53 inactivation in evasion of apoptosis and DNA repair is well established, but p53 also seems to be involved in chemo- and radioresistance [116,117]. Abe *et al.* have demonstrated that Hh signaling inhibits p53 activity by stimulating Mdm2 that promotes p53 ubiquitination and degradation. Inhibition of Hh signaling recovered p53 activity in breast cancer cells lines, leading to DNA damage-induced apoptosis [118].

### 3.4. Hedgehog Inhibitors in Combination with Chemotherapy

The treatment options for metastatic castration-resistant PCa (mCRPC) are very limited, with the first-line treatment being chemotherapy, *i.e.*, docetaxel possibly in combination with prednisone. However, inevitably, resistance to chemotherapy occurs in more than 50% of patients [119]. Currently, second-line therapies targeting the androgen receptor (AR) pathway are under investigation, since abundant evidence has shown that mCRPC still remains driven by AR signaling [120–122]. Recently, abiraterone, an inhibitor of CYP17, which plays a key role in the production of androgens, has been approved by the FDA as a second-line treatment of mCRPC [123]. Enzalutamide (MDV3100) is another promising agent targeting AR signaling currently under clinical investigation. In a randomized phase III clinical trial for patients with mCRPC after chemotherapy, enzalutamide improved overall survival [124].

The effectiveness of chemotherapy is challenged by distinct mechanisms that mediate drug resistance at cellular level, *i.e.*, genetic changes (mutations, amplifications, epigenetics) that influence drug uptake, metabolism or export, but also limited drug delivery due to the microenvironment plays an essential role [125,126]. As for the latter, the tumor vasculature is characterized by poorly organized, immature and leaky blood vessels resulting in impaired blood supply and thus impaired drug delivery [127]. Therefore, if the chemotherapeutic agents are unable to access all tumor cells within a solid tumor, these cells will survive and give rise to tumor recurrence. Olive *et al.* investigated the combination of Hh signaling inhibition and gemcitabine in a mouse model of pancreatic ductal adenocarcinoma [102]. Hh inhibition by the Smo inhibitor IPI-926 increased intratumoral vascular density, leading to better perfusion of the tumor thereby improving the delivery of gemcitabine to the tumor. Eventually, this resulted in an extended median survival, a transient stabilization of the disease and a significant decrease in metastases to the liver [102]. In addition, Bahra *et al.* also reported that combined treatment with cyclopamine and gemcitabine has synergistic effects on the reduction of tumor growth in pancreatic adenocarcinoma xenografts [103].

Emerging data suggest that cancer stem cells (CSCs) are the main source of clonogenic cells that give rise to tumor recurrence after chemotherapy [128–130]. CSCs consist of specific defense mechanisms against chemotherapy. For example, they contain a high level of ATP-binding cassette (ABC) proteins that acts as drug efflux pumps to actively remove harmful drugs out of cells, rendering them ineffective. Furthermore, CSCs contain multiple enzymes capable of drug metabolism and several DNA repair and anti-apoptotic proteins [131]. Several lines of evidence indicate that Hh signaling plays a key role in the regulation of cancer stem cells (CSCs). Hh signaling regulates the transcription of a number of genes implicated in cell fate determination and stemness features, *i.e.*, self-renewal and pluripotency [84,132]. A recent study by Nanta *et al.* has demonstrated that inhibition of Hh signaling with the Smo inhibitor NVP-LDE-225 (Erismodegib) down-regulates pluripotency-maintaining factors Nanog, Oct4, Sox2, c-Myc and thereby inhibits CSC tumor growth [65]. Moreover, Hh signaling promotes multidrug resistance (MDR) by increasing transcription of ABC transporter proteins ABCB1 and ABCG2 in different tumor types, including PCa [133–135]. Targeting Hh signaling in combination with chemotherapy could thus not only eradicate the bulk tumor cells, but also the chemoresistant CSCs responsible for repopulation of the tumor after chemotherapy.

Inhibition of Hh signaling could potentially reduce chemoresistance through other mechanisms (Figure 2) [136]. For instance, Narita *et al.* have demonstrated that inhibition of Hh signaling increases chemosensitivity of PCa cells. They have shown that inhibition of GLI2 significantly enhanced the effect of paclitaxel on PCa cells both *in vitro* as *in vivo* presumably through synergistic effects on apoptosis [64]. A study by Mimeault *et al.* demonstrated that co-targeting of the Hh and EGFR pathway in combination with chemotherapeutic agents results in increased anti-proliferative, anti-invasive and apoptotic effects on different metastatic PCa cell lines compared to the single or two-drug strategies, indicating that targeting both signaling pathways could enhance chemosensitivity [137]. Domingo-Domenech *et al.* have identified a docetaxel-resistant subpopulation in mCRPC that is characterized by elevated Hh and Notch signaling activity. Concomitant inhibition of both pathways resulted in depletion of this chemoresistant subpopulation, thereby abrogating tumor regrowth after docetaxel treatment *in vivo.* This effect is presumably due to the modulation of PI3K/AKT and Bcl2 by respectively the Notch and Hh pathway [138].

Despite these promising preclinical results, the combination of Hh inhibition and chemotherapy has not proven to be effective in clinical trials thus far. A phase II clinical trial testing the effect of IPI-926 in combination with gemcitabine has recently been closed early due to a decreased median survival in the combination group compared to the patient group treated with gemcitabine alone [139]. Currently, multiple phase I/II clinical trials are ongoing, testing the combination therapy of chemotherapy and Hh inhibition in several tumor types, including pancreatic, lung, gastroesophageal cancer and leukemia [77].

### 3.5. Hedgehog Inhibitors in Combination with Other Molecular Targeted Agents (MTAs)

In recent years, the focus of anticancer drug development has shifted from conventional chemotherapeutics, only affecting rapidly dividing cells, to more targeted agents that specifically interact with molecules essential for tumorigenesis. Numerous molecular targeted agents (MTAs) are being developed with higher efficiency and less side effects [140,141]. As mentioned above, the use of Hh inhibitors as single agents did not appear as successful as expected due to the frequent emergence of resistance. Combination of Hh inhibitors with other MTA, preferentially targeting a different tumorigenic pathway, could represent a promising alternative strategy.

#### 3.5.1. PI3K Inhibitors

Activation of PI3K/Akt signaling has been linked to tumorigenesis and resistance to both conventional and targeted therapies in a variety of tumor types, including PCa [142]. Moreover, in PCa, alterations in the PI3K/Akt pathway are commonly seen both in primary and metastatic lesions [143]. The RAS/MEK/ERK pathway is also frequently elevated in PCa samples [144] and seems to be correlated with progression to more advanced and androgen-independent PCa [145].

One of the potential mechanisms behind the development of resistance against Smo inhibitors is upregulation of PI3K signaling. This was illustrated by Buonamici *et al.* who found that PI3K target genes were upregulated in MB mouse models resistant to Smo inhibitor LDE225. Moreover, the combined treatment with Smo and PI3K inhibitors significantly delayed the development of resistance, although no differential effect was seen on tumor growth [82].

The PI3K/AKT and RAS/MEK/ERK pathways have been shown to non-canonically activate Gli1 in a Smo-independent manner [25]. The mechanism behind this is currently unknown, but mTOR/S6K1 could be the responsible link, since both AKT and ERK are able to activate mTOR. Wang *et al.* demonstrated that activated mTOR/S6K1 signaling promotes transcriptional activity and oncogenic function of Gli1 through S6K1-mediated phosphorylation of Gli1 at Ser84, thereby blocking the inhibitory function of Sufu. Moreover, co-treatment with mTOR/S6K1 and Smo inhibitors led to an increased inhibition of tumor growth *in vivo* in an esophageal xenograft mouse model compared to the single drug treatment [146].

Thus, Hh inhibition using a Smo inhibitor in combination with PI3K/mTOR inhibitor could increase the effectiveness of the treatment and potentially overcome resistance to Smo inhibitors.

#### 3.5.2. EGFR Inhibitors

Another attractive pathway in PCa is the ErbB/EGFR pathway. The ErbB signaling pathway is correlated with shorter survival and metastasis, and is also implicated in the development of androgen-independent PCa [147–149]. However, gefitinib, an EGFR inhibitor has failed to demonstrate efficacy in clinical trials in hormone-refractory PCa as a single agent [150]. Nevertheless, simultaneous inhibition of Hh signaling with cyclopamine and ErbB signaling with gefitinib or lapatinib yielded a synergistic effect on PCa cell growth *in vitro* [60].

Combined targeting of EGFR and Hh signaling by gefitinib and cyclopamine cooperatively improves the cytotoxic effects of docetaxel on metastatic PCa cells [151]. A phase I clinical trial is currently ongoing to test the effect of another EGFR inhibitor erlotinib hydrochloride in combination with GDC-0449 in patients with metastatic pancreatic cancer or solid tumors that cannot be removed by surgery [77].

#### 3.5.3. Androgen Deprivation Therapy

As stated above, resistance to androgen deprivation therapy is associated with increased Hh expression, which led to the assumption that Hh signaling could play an essential role herein. Suppression of Hh signaling in combination with ADT could therefore be a promising strategy to overcome this resistance. Currently, a phase I/II clinical trial is ongoing where the combination of an ADT (leuprolide acetate or goserelin) with or without GDC-0449 (preoperatively) in patients with locally advanced PCa is investigated [152].

#### 3.5.4. Others

Hh signaling inhibitors could be used in combination with many other molecular targeted agents that play an important role in the development and/or progression of PCa (angiogenesis inhibitors, other tyrosine kinase inhibitors, Notch inhibitors). Targeting multiple important signaling pathways at the same time should offer a solution to tackle the resistance problem. One important pitfall that has to be kept in mind, however, is the toxicity of the interaction between the different molecules and the effects on the normal tissue.

## 4. Conclusions and Future Perspectives

In recent years, the role of Hh signaling in PCa has actively been investigated and has shown to be involved in the development of PCa and progression to more advanced and castration-resistant disease. Targeting Hh signaling could therefore be a potential option for the treatment of PCa.

Clinical studies in patients with ligand-independent Hh activated tumors, *i.e.*, medulloblastoma and basal cell carcinoma have proven to be very successful. This even led to the FDA approval of GDC-0449 for treatment of locally advanced and metastatic BCCs. The effectiveness of Hh inhibition in ligand-dependent tumor types, such as PCa, is currently under clinical investigation, but preclinical data have shown that Hh inhibition has the potential to reduce PCa invasiveness and metastatic potential.

Unfortunately, resistance against Hh inhibitors is already seen in patients with MB and BCC during treatment with GDC-0449. Therefore, Hh inhibitors with another mechanism of action could be used as a means of second-line treatment. Another potential solution might be the combined treatment of Hh inhibitors with radiotherapy, chemotherapy or other molecular targeted agents, as described in this review. There are several indications that the Hh pathway could be a potential target for radiosensitization. Multiple lines of evidence have suggested a role for Hh activation in radioresistance. Furthermore, a number of target genes of Hh signaling are implicated in processes that influence the response to radiotherapy (Figure 2). Moreover, interactions between Hh signaling and other pathways known to be implicated in radioresistance (e.g., PI3K/Akt, RAS/MEK/ERK) have been described. Hh signaling could also contribute to chemoresistance by different mechanisms. First, by regulating cancer stem cells, which are known to be major contributors to chemoresistance. Next, Hh signaling promotes multidrug resistance by increasing transcription of important efflux pump proteins. Moreover, inhibition of Hh signaling seems to increase tumor perfusion and could thereby improve delivery of the chemotherapeutic agent to the tumor and also synergistic effects of Hh inhibition and chemotherapy on apoptosis have previously been described. Simultaneously targeting the Hh pathway and other tumorigenic pathways important in the development and/or progression of PCa, such as the PI3K/Akt, RAS/MEK/ERK, ErbB/EGFR and AR pathways, could also represent a promising therapeutic option for the treatment of advanced PCa. Preclinical data evaluating the combination of inhibitors of these pathways with Hh inhibition seem to be very promising. However, these promising result have not yet been translated into the clinic. Multiple clinical trials are currently ongoing to prove the efficacy of Hh inhibition in combination with chemotherapeutics and/or other MTAs in PCa.

In conclusion, there are many reasons to believe that Hh inhibition as monotherapy, but especially when used in combination with other therapies for the treatment of PCa, could be successful. However, more research into the exact mechanisms involved in Hh signaling (e.g., expression of primary cilia, mode of aberrant Hh signaling) is needed to gain more insight in the potential benefit of Hh-targeted therapy for PCa treatment. Furthermore, identification and validation of predictive markers is necessary to allow a better selection of those patients who will benefit from Hh targeting modalities. Moreover, more studies are needed to investigate possible interactions of the combined modalities, especially concerning toxicity. Although preclinical investigations regarding Hh inhibition for PCa have been favorable, the efficacy of Hh inhibition in PCa patients, either as a single agent or in combination with other drugs, has not been established yet. Currently, clinical studies testing the combination of Hh inhibitors with different chemotherapeutic agents and/or other MTAs are ongoing, but the combination with radiotherapy in the clinic is yet unexplored.

## Figures and Tables

**Table 1:** Overview of key associations between Hh signaling and clinicopathological parameters in PCa.

Study	# Tissue samples	Technique	Key findings	*p*-value
Tzelepi *et al.* [48]	141 PCa	IHC	Epithelial Shh, Smo and Ptch up-regulated in T *vs.* N	<0.001
	53 mPCa		Stromal Ptch, Smo and Gli1 down-regulated in T *vs.* N	<0.001
	119 N		Correlation Ptch1 and tumor grade/stage	<0.001
			Higher epithelial Ptch expression in metastasis *vs.* tumor	<0.001
			Correlation Hh signaling and proliferation (Ki67) and vasculogenesis (VEGF)	<0.001

Sanchez *et al.* [47]	239 PCa	IHC	Higher Shh expression in T (33%) *vs.* N (<1%)	<0.001
	15 HGPIN		Correlation Shh and proliferation (Ki67)	0.0141
	135 N		No correlation between Shh and other clinical parameters	

Fan *et al.* [34]	6 PCa	qPCR	No significant difference between Hh signaling in T *vs.* N	
	6 BPH			
	7 N			

Sheng *et al.* [45]	55 PCa	IHC	Hh signaling pathway frequently activated in advanced PCa	
	4 mPCa	qPCR	Correlation Ptch1 and Hhip with Gleason score and metastasis	
	55 N		Loss-of-SUFU frequently present in PCa	

Azoulay *et al.* [49]	275 PCa	IHC	In HNPC, correlation between epithelial Shh and Gleason, metastatic lymph nodes	<0.05
	(231 HNPC)	qPCR	Concomitant absence of stromal Shh and Dhh prognostic factor for PSA recurrence	0.01
	(20 HTPC)		Dhh expression up-regulated in epithelial HTPC and HRPC *vs.* HNPC	<0.0001
	(24 HRPC)			

Kim *et al.* [50]	155 PCa	IHC	Correlation between Shh, Ptch, Smo, Gli and Gleason score	<0.01
	155 N	qPCR	Shh independent prognostic factor for PSA recurrence	<0.001

Karhadkar *et al.* [1]	12 PCa	qPCR	Shh and Ihh present in all prostate samples	
	15 mPCa		PTCH1 and GLI1 mRNA expression tenfold higher in metastatic *vs.* tumor tissues	
	12 N			

Efstathiou *et al.* [54]	79 PCa	IHC	Up-regulated Hh signaling (Gli1, Gli2, Smo, Shh) after ADT or ADT with chemotherapy	<0.05
	26 (ADT)		Nuclear pAKT increased	<0.001
	27 (ADT + CT)		Epithelial Bcl2 increased after combination treatment	<0.01
	27 (Untreated)			

Ibuki *et al.* [55]	210 PCa	IHC	Dhh expression up-regulated after long-term ADT	
	(44 ST-ADT)		Shh expression elevated in HRPC specimens	
	(76 LT-ADT)			

Abbreviations: RP, radical prostatectomy; PCa, prostate cancer tissue; N, normal tissue; mPCa, prostate cancer metastasis; HGPIN, high-grade prostatic intraepithelial neoplasia; BPH, benign prostate hyperplasia; HNPC, hormone-naïve prostate cancer; HTPC, hormone-treated prostate cancer; HRPC, hormone-refractory prostate cancer; CT, chemotherapy; ST-ADT, short-term androgen deprivation therapy; LT-ADT, long-term androgen deprivation therapy; IHC, immunohistochemistry; qPCR, quantitative real-time polymerase chain reaction.
